# 3D Printing Breast Tissue Models: A Review of Past Work and Directions for Future Work

**DOI:** 10.3390/mi10080501

**Published:** 2019-07-27

**Authors:** Chantell Cleversey, Meghan Robinson, Stephanie M. Willerth

**Affiliations:** 1 Doctor of Medicine (MD), Faculty of Medicine, University of British Columbia, Vancouver, BC V6T 1Z3, Canada; 2 Department of Urological Sciences, Vancouver Prostate Centre, Vancouver, BC V6H 3Z6, Canada; 3 Department of Mechanical Engineering and Division of Medical Science, University of Victoria, Victoria, BC V8W 2Y2, Canada

**Keywords:** breast reconstruction, 3D-printing, adipose tissue, biomaterials, stem cells, tissue engineering, drug screening

## Abstract

Breast cancer often results in the removal of the breast, creating a need for replacement tissue. Tissue engineering offers the promise of generating such replacements by combining cells with biomaterial scaffolds and serves as an attractive potential alternative to current surgical repair methods. Such engineered tissues can also serve as important tools for drug screening and provide in vitro models for analysis. 3D bioprinting serves as an exciting technology with significant implications and applications in the field of tissue engineering. Here we review the work that has been undertaken in hopes of generating the recognized in-demand replacement breast tissue using different types of bioprinting. We then offer suggestions for future work needed to advance this field for both in vitro and in vivo applications.

## 1. Introduction

Breast cancer is the second leading cancer by incidence worldwide, with over two million cases estimated in 2018 according to the World Health Organization (WHO). It is also the most commonly occurring cancer amongst females and the leading cause of cancer-related death amongst females [1]. The American Cancer Society reports that breast cancer carries a promising 5-year survival rate of 90% [2]. However, a 2018 systematic review concluded that depending on the number of years post-primary breast cancer, 1.9% to 11.1% of patients curatively treated will experience ipsilateral breast cancer recurrence [3]. Ultimately, an estimated 28–60% of breast cancer cases require a mastectomy where surgery is performed to remove the breast [4]. As a consequence, a high demand for breast reconstructive surgery exists, creating a great deal of economical cost to the healthcare system as well as psychological cost to the patient [5]. Current options for breast reconstruction include implant- or autologous-based “flap” methods [6]. In the early 1990s, lipofilling became a well-established procedure, where fat from the patient is retrieved using liposuction and reintroduced by injection in areas requiring shape and contour modification [7]. Recently, this practice, often referred to as fat grafting, has been adopted on a larger scale with aims of reconstructing the entire breast, but faces the challenge of significant volume loss after implantation [8].

Breast implants consist of sacs filled with saline, silicone gel, or a mixture of both [9]. While implant-based reconstruction entails a relatively short surgical and recovery time, implants have an expected lifespan of 10–20 years, as stated by the American Society of Aesthetic Plastic Surgery and the American Society of Plastic Surgeons [10]. Thus, patients with implants will often need additional surgery later in life due to the implant lifespan. Most strikingly, these patients face a 40% re-operation rate within 5 years of reconstruction from complications such as infection, seroma or hematoma development, asymmetrical or malposition outcomes, capsular contracture caused by the body’s foreign body immune response, and implant rupture or deflation [11]. Autologous reconstruction generates a replacement breast from tissue (skin, fat, muscle) taken from elsewhere on the patient’s body. The use of patient-derived tissues contributes to a more texturally natural aesthetic and avoids the foreign body immune response [12]. This method requires a more complex procedure, significant time and expense, and it can result in muscle weakness and hernia formation at the tissue donor site [11].

Fat grafting has been of interest to surgeons and researchers for hopes of overcoming these limitations, because this process injects autologous adipose tissue into the breast region without the need for invasive tissue grafting [11]. Unfortunately, the newly injected adipose tissue lacks vasculature, which results in a volume loss of 20–70% over time [11,13]. It can lead to harmful effects like long-term inflammation and progressive calcification. Thus, this method has its own drawbacks to consider when evaluating options for breast reconstruction. Following the limited success of fat grafting, researchers have tried enriching the fat-graft tissue with autologous adipose-derived stem cells (ASCs) beyond the naturally occurring concentration. In 2008, Yoshimura et al. pioneered a technique known as cell-assisted lipotransfer (CAL), in which autologous fat grafts are enriched with autologous ASCs [14]. ASCs are collected from the isolated stromal vascular fraction of physically and chemically processed adipose tissue and then added back into adipose tissue destined for patient injection. ASCs are adult mesenchymal stem cells found in several tissues throughout the body but found in particularly high abundance in adipose tissue. These cells possess multilineage potential, including the ability to differentiate into adipocytes, and they can also self-renew, making them ideal for adipose tissue regeneration and angiogenesis [13,15,16]. Several studies have been employed to ascertain the benefits and drawbacks of this method. A review published in 2016 by Arshad et al. reported overall positive results in 11 studies; however, they also note an absence of controls within 8 of these studies [17]. Thus, findings on whether enriching fat grafts with ASCs serves as an improvement over conventional fat grafting is currently ambiguous.

Furthermore, the lack of long-term follow-ups in these studies makes it difficult to conclude their overall safety and their contribution to breast cancer recurrence. A study performed by Eterno et al. found that ASCs contribute to metastasis and proliferation of c-Met expressing breast cancer cells [18]. Likewise, a study by Sakurai et al. suggested that cytokine production by ASCs had the potential to stimulate breast carcinoma cell growth by upregulation of S100A7 expression [19]. However, these findings were not validated in clinical trials and reports of tumor recurrences after breast cancer reconstruction have been very low to date. Thus, the true risk of using ASCs in breast cancer reconstruction is still debatable [15,20]. Nevertheless, there is research being done to mitigate this potential risk by testing ASCs as anti-cancer drug delivery systems [21]. One such study by Scioli et al. showed that ASCs can uptake and release the chemotherapeutic agent Paclitaxel (PTX) to suppress CG5 breast cancer cell proliferation and survival both in vitro and in vivo in mice, suggesting that ASCs may have therapeutic potential in suppressing breast cancer recurrence [22]. 

Tissue engineering can address the limitations of the predominantly used autologous tissue and implant-based reconstruction methods. This process combines cells with synthetic or natural structural and biochemical components, which provide a microarchitecture for cell attachment, growth and differentiation into a complete tissue [23]. Tissue engineering has the advantages of using patient-derived cells, making it autologous, while also supplying the necessary architecture and vasculature to support long-term stability and viability. Structural extracellular matrix components are typically biodegradable, allowing them to be replaced over time by cell-secreted extracellular matrix proteins from the regenerating tissue. Furthermore, these structural components can be designed to release signaling molecules as they degrade, allowing for precise control over the directed differentiation of the cells within the construct [24].

Generating adipogenic cells from ASCs requires specific conditions in terms of scaffold stiffness, pore size, microstructure pattern, and surface nanotopography [25]. Therefore, a successful scaffold will closely mimic that of natural adipose tissue, and will therefore include the extracellular matrix components found in natural breast tissue; namely, collagen I, hyaluronan, laminin, and fibronectin. Illustrating the powerful effect of these extracellular matrix components to guide the differentiation of breast tissue, Sokol et al. designed a scaffold using collagen, laminin, fibronectin and hyaluronan and seeded it with primary human breast epithelial cells [26]. They reported that these scaffolds directed the adoption of lobular and ductal tissue morphologies and promoted correct organization of luminal, basal and stem cells in 26% of the tissues, whereas collagen-only scaffolds had no effect. Approximately 5% of the regenerated tissues successfully matured and became functional expressing hormone responsive estrogen and progesterone receptors, illustrating promising results for future research. In contrast, Findlay et al. tested the use of hard acrylic chambers containing vascular pedicle grafts to promote infiltration of host adipose tissue in a pig model. These chambers regenerated clinically relevant volumes of adipose tissue in the pig model. However, in the first and only human trial using these methods by the Morrison et al. group, results predominantly yielded mainly fibrotic tissues [27,28]. Research involving ASCs has shown the most promise in breast tissue engineering, but multiple challenges remain in this field. Finding a suitable structural matrix to support ASC growth and differentiation while preventing tissue damage as regeneration occurs remains a predominant and fundamental challenge. Secondly, generating a clinically sufficient volume of adipose tissue that proves to be sustainable is vital, and poses a significant challenge because environmentally-dependent angiogenesis is required to provide the necessary vasculature for tissue survival [11].

3D bioprinting serves as a promising strategy to address these challenges, due to its ability to generate highly controlled complex 3D architectures using automation. The recent development of novel 3D bioprinting technologies allows for the creation of precise and reproducible cell-laden architectures that mimic the target tissues or organs. 3D bioprinting, an additive manufacturing technique, uses a computer-generated design to build a 3D structure by depositing materials in a layer by layer fashion, with resolutions on the order of microns. Because 3D bioprinted tissue can be made to reflect the micro-organization of natural tissue, appropriate cell-cell interactions that mediate proper tissue development and function can occur. Furthermore, the automated nature of 3D-printing offers the ability to upscale printed structures to produce clinically relevant volumes and sizes. Such 3D printed tissues can also serve as important tools for drug screening [29]. A 3D-bioprintable design is particularly advantageous for breast reconstruction, because it allows for the incorporation of vascular networks, a mechanically supportive framework, patient-specific sizes and shapes, and even a nipple-areola complex [25,30]. 

## 2. Materials and Methods 

### 2.1. Review Methods

We scanned online databases for existing literature and news on breast tissue regeneration via 3D-printing. These databases included Medline Ovid, Google Scholar, PubMed, and Google searches of ongoing work discussed in press releases. Key words used in online databases included breast tissue, 3D, 3-dimensional, 3D-printing, 3D-bioprinting, reconstructive surgery, breast reconstruction, adipose tissue, implant, regeneration, mastectomy, tissue engineering. Multiple broad titles were used in the Google Search bar to pick up any ongoing research in the press that is related to our topic. We then selected relevant articles to include if they used 3D-printing in attempt to generate breast tissue. There were no limitations on date of publication for literature found.

This review aims to summarize the work that has been done to date using 3D-printing to generate breast tissue for both tissue engineering and drug screening applications. These studies are all pre-clinical and the majority were undertaken by two groups, the Pati group and the Chhaya group, both of whom have reported success in regenerating adipose tissue in animal models. We then offer our suggestions for future directions for the field.

### 2.2. Bioprinting Methods

**Figure 1:**
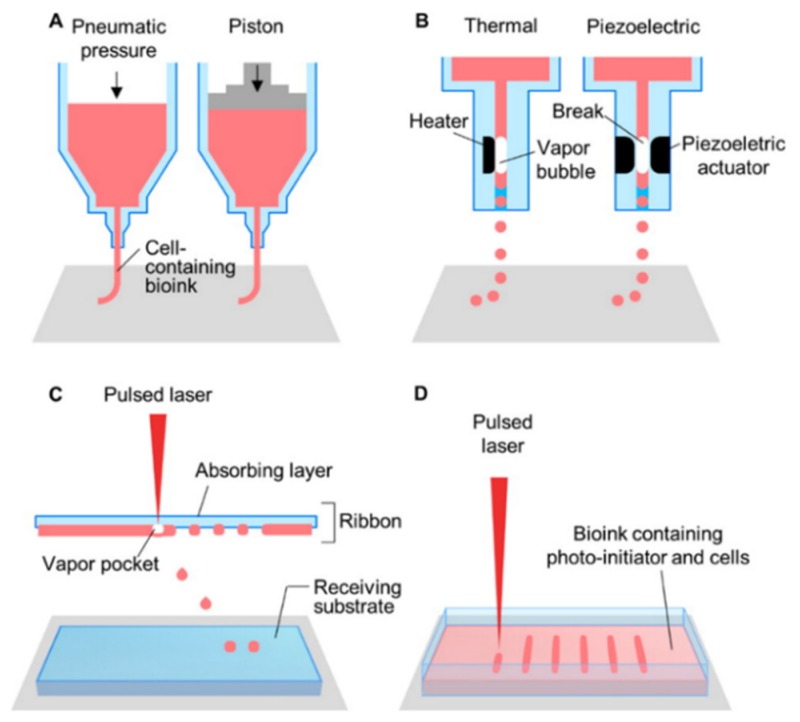
Schematic illustrations of (**A**) microextrusion, (**B**) inkjet, (**C**) laser-assisted printing, and (**D**) stereolithography techniques. Reprinted with permission from [31]. Copyright 2016 American Chemical Society.

Bioprinting strategies fall into four categories: Extrusion-based, inkjet, laser-assisted, and stereolithography-based (Figure 1, Table 1). Each technique presents with its own advantages and limitations (Table 1) with a common aim of minimizing cell damage. The preferred bioprinting technique will be dependent on the tissue of interest and the scalability required by the application. A technique that offers fine resolution to the degree of vasculature size, high speed production, and low costs to enable the generation of the large volumes, is most appropriate for a softer, larger tissue such as breast tissue. 

**Figure 2:**
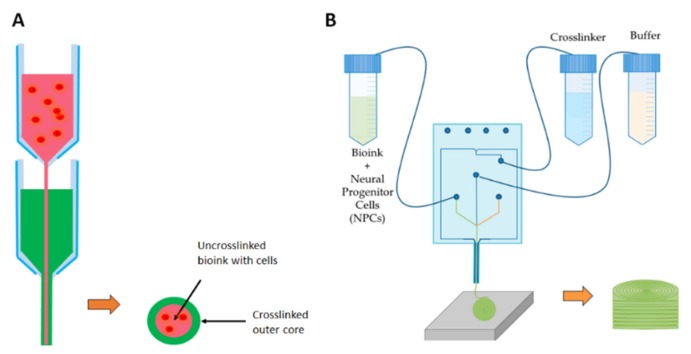
Schematic showing advances in extrusion-based bioprinting. (**A**) Core-shell nozzles encase an inner cell-laden bioink within a protective cross-linked bioink to reduce shear-stress induced cell death. (**B**) An example of a microfluidic printhead design in which a cell-laden bioink is introduced to the crosslinker as the channels merge, allowing for crosslinking to take place moments after extrusion, thereby greatly reducing shear stress-induced cell death [39]. This figure is being reprinted under a Creative Commons BY 4.0 license.

Extrusion-based printing (Figure 1A, Table 1) generates either pneumatic, piston- or screw-driven continuous pressure to push a bioink through an orifice. This provides the ability to produce a cell-laden, high viscosity, continuous fiber of bioink that can build a complex 3D structure [32,33]. Albeit, slow speeds are required to avoid cell damage, and the best resolution reached is ~100 µm. Higher viscosity bioinks result in greater shear stress on cells compared with low viscosity, contributing to reduced cell viability [32,34]. Recent advancements in extrusion-based printing, including the incorporation of melt electrowriting, microfluidics, and the development of a core-shell nozzle have begun to offer improvements in terms of resolution and cell viability. Melt electrowriting is based on the principle of electrospinning: a polymer solution or melt is electrostatically drawn through a positively-charged needle onto a grounded collector to produce incredibly fine fibers [35]. In melt electrowriting, extrusion-based printing directly writes fibers into precisely stacked 3D constructs, generating fibrous microarchitectures closely mimicking those of a natural extracellular environment, with resolutions between 10–100 µm. Unfortunately, this method cannot currently be combined with cell-laden bioinks due to the high temperature (78 °C) and high voltage (10–12 kV) involved in the process, therefore requiring cell seeding post-printing. Core-shell printing nozzles (Figure 2A) generate a stream of bioink wherein an inner core is laden with cells while an outer core is subjected to crosslinking, becoming a protective shield to the cell-laden inner core while improving the overall mechanical strength of the resulting construct [32,33]. This process greatly improves the viability of the cells in the inner core, as the outer core absorbs the majority of the shear stress. Further improving both viability and resolution is the design of microfluidic printheads (Figure 2B). The use of microfluidics in place of conventional nozzles reduces shear stress by enabling separate bioink and crosslinker streams to merge at the point of extrusion. This multi-component design presents opportunities for printing multiple bioinks from a single printhead and enables micro to nanometer resolution matching sizes of blood vessels, nerves and muscle units [36].

Inkjet printing (Figure 1B, Table 1) involves dispensing 1–100 µL droplets of a cell-laden bioink in a precise manner to produce a pattern [32]. Droplets are dispensed by means of either thermal heating or piezoelectric pressure waves. Thermal heating technique (Figure 1B) uses an external thermal element located on the nozzle, reaching temperatures between 100–300 °C to generate an internal vapor bubble which pushes a droplet through the orifice [32,33,34]. With a similar concept, piezoelectric inkjet printers (Figure 1B) use bilateral piezoelectric elements to generate a mechanical pulse via acoustic waves to force a droplet through the orifice [32,33]. Inkjet printers can achieve resolutions of 50 µm, are low cost and widely available [32,33]. However, the ability to generate complex 3D architectures is limited, because the often-required high-viscosity bioinks for such structures commonly clog the orifice. High viscosity bioinks can also impede droplet formation by dampening the acoustic pressure waves, specifically in piezoelectric inkjet printers. In addition to the structural limitations, reduced cell viability may result from heat, acoustic pressure or shear stress (i.e., a small orifice). Although the heat is localized, it is unlikely a perfect system and mammalian cells readily shift towards cell death at a critical temperature of 43 °C, well below temperatures used in thermal inkjet printing. The frequency (15–25 kHz) of acoustic waves used in piezoelectric inkjet printing is within the range of sonification known to cause cell membrane damage and lysis [37,38,39]. It is known that cell behavior is influenced by mechanical forces through mechanoreceptor integrins, pericellular tethers, focal adhesions, ion channels, cadherins, connexins, and membrane lipid rafts. Yet, while it is unclear why internal strain leads to cellular death, is it known that shear stress causes excessive internal strain contributing to cell death [32].

In contrast to extrusion-based and inkjet printing modalities, laser-assisted printing (Figure 1C, Table 1) is a completely nozzle-free method. Instead, a multilayered “ribbon”, composed of an absorption layer and a bioink layer, beads droplets of bioink upon laser pulsations. The laser pulse heats the metal layer, thereby generating a vapor bubble that propels bioink onto a receiving substrate [32,40]. This effect is known as laser-induced forward transfer (LIFT), and eliminates the risk of shear-stress-induced low cell viability. However, similar to thermal inkjet printing, the risk of heat-induced cell death exists due to laser irradiation [32,40]. A major advantage is the ability to print viscous or even solid bioinks with resolutions of 1–50 µm [34]. However, the advantages are offset by its slow printing time, high cost and limited scalability.

Stereolithography (Table 1) is another nozzle-free printing technique wherein a vat of bioink is polymerized at its uppermost layer, then dipped and polymerized again; with the process being repeated to produce a 3D structure. The bioink must be photosensitive, as light energy is the crosslinking agent; therefore its use is restricted to photo-curable bioinks. This introduces the problem of cell death due to cytotoxic photo-initiators and UV irradiation [32,33,34]. Furthermore, exposure to UV radiation can induce mutations leading to cancer, so this technique is not suitable for printing stem cells. Advances are being made to replace UV light with visible light to avoid this complication. A major advantage of photo-curable bioinks is that they generate strong covalent bonds to allow mechanically strong 3D architectures to be realized, with impressive resolutions between 5–300 µm. Although a fine resolution on scale with vasculature size can be achieved, producing hollow architectures remains problematic, as the uncured bioink in the vat is free to drip into the previously cured layer, filling in hollow structures.

When choosing a bioink, there are three fundamental questions that need to be addressed. Firstly, what are the characteristics of the tissue being printed? It is important to delineate whether the tissue is hard or soft (Young’s modulus as an objective measure), and the extent of vascularization that the tissue naturally relies on. For example, cartilage is hard and requires minimal blood supply in comparison to highly vascularized, soft adipose tissue [40]. Secondly, because structure strength, resolution, and shape are dependent on the printing technique, one must consider which technique would be compatible with the physical properties of the bioink and expected structural outcomes. As previously mentioned, the main options include extrusion-based, inkjet, laser-assisted, and stereolithography (Figure 1, Table 1). Thirdly, when considering a bioink material, it is important to confirm that it is biocompatible, biodegradable, and does not cause any toxic effects, including from breakdown products. 

Basic bioink requirements are both biological and material-based. In terms of biological requirements, the bioink should be biocompatible and provide sufficient permeability of oxygen, nutrients and metabolic wastes [41], and also contain biochemical cues to promote cell adhesion, migration and proliferation [42]. In terms of material qualities, printability and the mechanical integrity of the structure post-print are determined by viscosity, surface tension, cross-linking properties, gelation kinetics, degradation rates, and cell encapsulation densities [43,44]. For example, in inkjet printing, droplet integrity is necessary for precision, and depends on droplet size, density and surface tension. In contrast, extrusion-based printing relies on the shear-thinning character of the bioink to facilitate extrusion through the nozzle at a low pressure, and to then self-heal to promote the stability of the construct. 

Degradation of the bioink over time is key to allow the cells to replace the bioink with their own extracellular matrix over time, promoting integration of the constructs in vivo [42,43]. Furthermore, the timing of degradation should coincide with the integration of the constructs. The degradation rate can be dependent on whether the biomaterials used are natural or synthetic, and the cellular density [42,43]. Studies have shown that constructs seeded with high cellular densities exhibit accelerated extracellular matrix remodeling [44].

Bioinks can be either natural or synthetic. Natural bioinks are derived from plants and animals, so typically contain bioactive components, are biocompatible, degradable and structurally similar to native extracellular matrix [42]. Examples include agarose, alginate, chitosan, gelatin, fibrin and decellularized extracellular matrix. Synthetic bioinks can also be biocompatible, but often must be altered to contain bioactive cues [41,42]. Compared to natural bioinks they are mechanically more stable, and their properties more easily controlled. Examples include pluronic and poly(ethylene glycol). Bioinks can also be scaffold-free, simply containing cell aggregates within a carrier solution. In these bioinks, cell-cell interactions guide the self-assembly of densely packed cellular aggregates into tissues [42]. However, this often results in low viability due to mechanical stress on the cells during printing [44]. 

**Figure 3:**
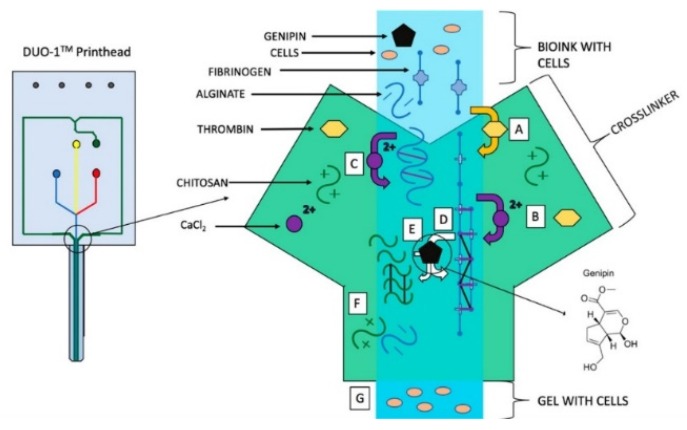
Reaction between the bioink and cross-linker in Aspect Biosystems’ DUO-1 Printhead. (**A**) Thrombin cleaves fibrinogen to form fibrin monomers, which aggregate to form protofibrils. (**B**) Calcium chloride stabilizes fibrin and promotes polymerization. (**C**) Calcium chloride crosslinks alginate. (**D**) Genipin cross-links fibrin. (**E**) Genipin cross-links chitosan. (**F**) Alginate and chitosan interact due to polarity. (**G**) Printable gel enters the nozzle of the printhead and is subsequently deposited on the print bed. Reprinted with permission from [46]. Copyright 2019, American Chemical Society.

The needs of each bioprinting application are different, and as such, bioink design is often unique and complex, involving several materials. To illustrate the extent of the complexity that can be involved, we look at one example provided by Abelseth et al., who designed a bioink for printing human induced pluripotent stem cell-derived neural aggregates using a syringe-pump driven microfluidic printer [45]. This group chose to use a microfluidic printhead with separate channels for bioink and crosslinker to enable non-viscous flow during extrusion, protecting the cells from shear-induced stress and its associated effects of cell death and premature differentiation. This method also ensured consistent crosslinking such that the resulting stiffness and porosity of the structure were uniform, as those properties can also influence differentiation. The bioink itself incorporated fibrin, alginate, chitosan, calcium chloride, thrombin, and genipin (Figure 3). Fibrin is a natural biomaterial derived from blood, and is often used as a sealant in surgery [43]. Its precursor form, fibrinogen, is cleaved by thrombin to initiate polymerization into a fibrous microarchitecture resembling that of extracellular matrix. Fibrin is biocompatible, promotes cell attachment, and can promote neural differentiation from progenitor cells [46]. Unfortunately, it is mechanically weak, leading to short degradation times and a lack of printability due to slow gelation kinetics. Chitosan, a natural polysaccharide derived from crustaceans, was incorporated to improve the stability of the bioink; while the addition of the genipin, a crosslinker found in plants, was used to improve its integrity over time by further crosslinking the amine groups of fibrin and chitosan [45,47]; and lastly, alginate was used to make the bioink printable. Alginate is a natural polysaccharide derived from seaweed with excellent biocompatibility and gelation properties, but no bioactive components. The addition of calcium ions in the crosslinker rapidly polymerized the alginate component in the bioink, providing the viscosity needed to build a 3D structure. This bioink was used to successfully generate mature motor neurons from human induced pluripotent stem cell-derived neural progenitors [48].

## 3. Results

### 3.1. Using Computer Aided Design (CAD) and Computer Aided Manufacturing (CAM) Technology to Assist Surgeons with Breast Reconstruction

Computer aided design (CAD) and computer aided manufacturing (CAM) take advantage of computational technology to improve the design of the intended products. Melchels et al. used CAD/CAM technology to create life-size, patient-specific customized molds to aid in autologous breast reconstruction [49]. They performed laser scanning to generate the digital model of the patient’s breasts, which was then transformed to a physical mold using 3D-printing. The surgeons then used the mold intra-operatively during autologous free flap reconstruction to guide the contour and positioning of the free flap. A case study was completed with three patients, in which it was concluded that patient satisfaction of shape and symmetry was higher using these methods compared to the control group. The same technology was then used to generate customized porous scaffolds from non-biodegradable acrylonitrile butadiene styrene copolymer, as a proof of principle for the future use in tissue engineered breast reconstruction. This work indicates the strong potential for CAD to generate patient specific tissue shapes when used in combination with 3D printing. 

### 3.2. Generating a Bioink from Adipose Tissue to 3D-Bioprint an Implantable Porous Cell-Laden Structure Resembling Breast Tissue 

The Pati group isolated tissue-specific extracellular matrix proteins, broadly termed decellularized extracellular matrix (dECM), to generate a bioink for 3D-bioprinting a cell-laden structure using human adipose-derived stem cells (hASCs) [50]. They decellularized the adipose tissue using physical, chemical and enzymatic methods, followed by peracetic acid and ethanol washes as a disinfection step. A DNA quantification assay was used to evaluate the efficiency of decellularization, which proved to reduce cellular content by ~98%. Hematoxylin and eosin (H&E) staining was also employed to evaluate the degree of decellularization, and proved to confirm the absence of cells and cellular content. The group estimated collagen and glycosaminoglycan (GAG) content pre- and post-decellularization using the hydroxyproline and dimethylmethylene blue assays, respectively, as a means of assessing extracellular matrix (ECM) loss from the decellularization process. Analyses showed collagen content did not significantly increase, while GAG content decreased nearly 50%. The group attributed the detected elevation in collagen as a result of an increased collagen proportion to overall tissue material after cell and fat loss from the decellularization process. They noted that the natural organization of ECM fibers was lost during processing and that non-identified proteins were present as indicated by sodium dodecyl sulfate-polyacrylamide gel electrophoresis. They employed this dECM-based scaffolding as a temperature-dependent bioink, which was kept in a cooled liquid state before printing to allow the incorporation of ASCs as a liquid cell suspension, and then gelled, due to a temperature rise upon printing, in order to build a cell-laden 3D structure providing a similar microenvironment to that of natural adipose tissue. The group solubilized their dECM to a 3% concentration and adjusted it to physiological pH, prior to adding cells to generate their bioink. Following pH adjustment, the process was completed while maintaining a temperature of 10°C to prevent gelation. The material was seen to remain in a solution state below 15 °C and become a solid gel when incubated at 37 °C for 30 min.

**Figure 4:**
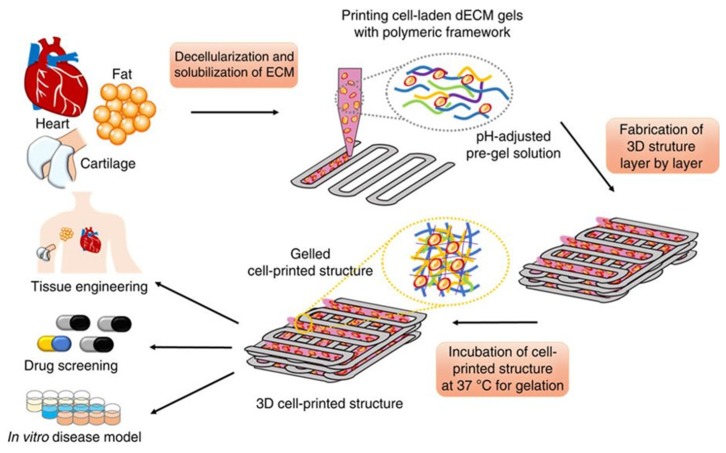
Schematic elucidating the tissue printing process using decellularized extracellular matrix (dECM) bioink taken from [50]. This figure is being reprinted under a Creative Commons BY 4.0 license.

They then 3D-bioprinted an open, porous, cube shaped construct using a hybrid extrusion technique, whereby polycaprolactone (PCL), a Food and Drug Administration (FDA)-approved biodegradable thermoplastic polymer, provided the framework for the cell-laden bioink that followed from a second extrusion print head (Figure 4). hASCs were chosen as the cell source in the cell-laden bioink, and a high density of 5 × 10^6^ cells/mL was successfully used. The group highlights the importance of a high density bioink for promotion of adipogenic differentiation, as they found that the high density promotes the stem cells to become round and halt cell growth, thereby promoting differentiation to adipocytes. The PCL framework created porosity, enabling oxygen and nutrient supply to cells encapsulated by the bioink. A fine PCL line width of 100 µm was chosen to reduce stiffness, and the cell-laden bioink was laid down in alternating gaps between PCL lines for up to 10 layers. They emphasized minimizing shear force during extrusion and concluded that the 2 s^−1^ shear rate used did not increase apoptotic levels compared with non-printed cells. The cell-laden bioink temperature was maintained below 15 °C during extrusion, and upon completion of printing the structure was gelled in a 37 °C humidified incubator for 30 min. The printed structure remained stable while in culture for at least 14 days, and cell viability testing proved >90% remained viable. Cell-cell connections were also observed as early as 24 h post-print. To identify and quantify adipogenic differentiation within the printed construct, tissue-specific gene expression was targeted. Expression of the master adipogenic regulatory genes peroxisome proliferator-activated receptor gamma (PPARγ) and CCAAT/enhancer-binding protein alpha (C/EBPα), and the early adipogenic marker lipoprotein lipase (LPL) were found to have increased levels in their formulated dECM-bioink compared to controls collagen and alginate, and other commonly used bioinks. These adipogenic markers were observed to increase over time, suggesting that their bioink promoted an adipogenic differentiation. As shown in Figure 1, these newly engineered breast tissue models can be used as both tissue replacements and as a valuable tool for drug screening applications. 

As an extension to the above study, they then completed an in vivo study using nude mice where their 3D-bioprinted constructs were subcutaneously implanted [51]. They used the same biomaterials and 3D-bioprinting methods, however they printed a 10 mm × 5 mm dome-shaped construct, instead of the previous cube construct. The dome shape was chosen to eliminate possible stress-induced tissue degeneration at the high stress corner regions. They also investigated the effect of adding exogenous adipogenic differentiation factors to their 3D-bioprinted constructs for two weeks of in vitro culture. Their findings show that exogenous factors promoted higher levels of adipogenic gene expression (PPARγ, C/EBPα, LPL), although a reasonable amount of gene expression was observed without, pointing to the ‘adipo-inductive’ effect attributed to the bioink. They also investigated the ability of their 3D bioink to sustain viability in culture, assessing the top layer and center region of the construct on days 1 and 14. Cell viability was concluded to be stable, at >90% on both days for the top layer; however, viability for the center region dropped from ~93% on day 1 to ~84% by day 14. For their in vivo study, the group subcutaneously implanted four different construct types in nude mice: A PCL scaffold (no dECM or hASCs), a PCL-dECM hybrid (no hASCs), a cell-laden PCL-dECM construct, and an injectable cell-laden dECM. They harvested tissue for analysis at weeks 2, 4 and 12. The PCL scaffold did not show any relevant results, and the injectable cell-laden dECM progressively dissipated, becoming undetectable at 12 weeks. In contrast, the PCL-dECM constructs, with and without hASCs, showed positive adipogenic results. Functional blood vessels were histologically visualized as early as 2 weeks. Type IV collagenous architecture was noted at 2 weeks and integrated with host tissues by week 12, demonstrating successful tissue remodeling. PPARγ-positive cells were detected at 2 weeks, with an increase by 4 weeks confirming adipogenic differentiation. Inflammatory cell infiltration was noted at the periphery of both construct types at 2 weeks but decreased by 4 weeks. Interestingly, their dECM-bioink printed construct generated similar levels of adipogenesis with or without hASCs, showing that their bioink was able to co-opt host tissue for regeneration. Overall, this work shows the potential of using dECM-bioink in combination with ASCs as a way to engineer breast tissue. 

### 3.3. Implanting Large Polymer Scaffolds Combined with Autologous Adipose Tissue in Minipigs 

**Figure 5:**
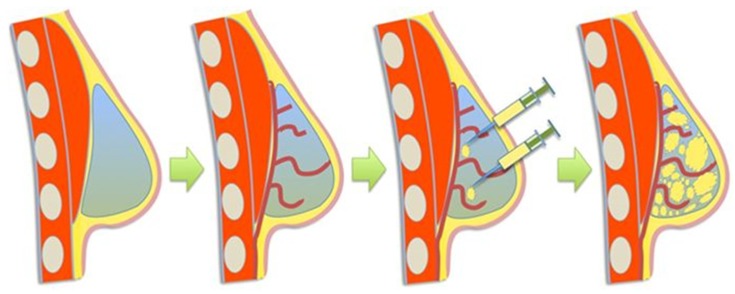
Schematic showing the process of pre-vascularization followed by delayed fat injection taken from [52]. This figure is being reprinted under a Creative Commons BY 4.0 license.

Chhaya et al. are the first group to generate and sustain a large volume of adipose tissue using a 3D-bioprinted biodegradable scaffold [52]. They employed laser scanning to acquire a patient-specific model for their breast tissue scaffold. Using fused deposition 3D-printing, the 3 cm^3^ breast-shape scaffold was constructed using poly(ᴅ,ʟ)-lactide polymer, a biodegradable plastic often used in medical device fabrication. The scaffold was designed to have pore sizes of >1 mm and was then seeded with human umbilical cord perivascular cells to support the generation of blood vessels. It was cultured under static conditions in vitro for 4 weeks, followed by 2 weeks in a biaxial rotating bioreactor. The constructs were then further seeded with human umbilical vein endothelial cells and subcutaneously implanted in immunocompromised rats for 24 weeks. Angiogenesis was seen throughout the entirety of analyses, as was host adipogenesis, with adipose tissue formation observed to make up ~81% of the volume by week 24. Following the success of their mouse model, this group then completed a second in vivo study using two female immunocompetent minipigs, placing 6 × 75 cm^3^ scaffolds between the breast and pectoral muscle of each. Rather than seeding the constructs with perivascular or endothelial cells, they sought to stimulate regeneration by implanting a scaffold that would receive a small volume of lipoaspirate injection 14 days later, thus introducing a novel pre-vascularization technique that uses the patient’s own body as a bioreactor (Figure 5).

The rationale was that the implant would stimulate blood clot formation within the construct, leading to angiogenesis formation—peaking at 14 days, the optimal time for lipoaspirate injection. These 3D-printed scaffolds were made using medical grade PCL instead of the previously used poly(ᴅ,ʟ)-lactide polymer. The group implanted the structure alone, with lipoaspirate, and with a delayed lipoaspirate injection at day 14. The structure alone proved to induce primarily vascularized connective tissue, while the immediate lipoaspirate-containing scaffold and the delayed lipoaspirate-containing scaffold demonstrated a substantial amount of adipogenesis. The delay in lipoaspirate addition was thought to allow pre-vascularization in the construct, and these conditions produced the greatest change with a six-fold increase in adipose tissue generation from the initial injection volume over the 24-week period. Although the pre-vascularization group proved most effective with a six-fold increase versus a five-fold increase in the non-delayed lipoaspirate group, no statistical significance was found between these two groups implying the two groups were not significantly different. The pre-vascularized and non-delayed groups were estimated to contain approximately 28 cm^3^ and 24 cm^3^ of adipose tissue respectively, at the 24-week mark. Native breast tissue (control) has approximately 45% adipose tissue to a given tissue area. Both the pre-vascularized and non-delayed groups were assessed to have a similar adipose tissue percentage to that of the control (~47% and ~40% respectively), the difference being statistically insignificant. All groups showed substantial neovascularization, and although the blood vessel density was greatest in the pre-vascularization group, the difference was statistically insignificant, even compared with the control. Adipocyte surface area measured in the range of 100–700 µm^2^ for all groups; however, the pre-vascularized group contained a substantial amount of adipocytes, measuring greater than 800 µm^2^. To put this into context, native breast tissue contains adipocytes with surface areas in ranges 100–200, 300–400, and 500–600 µm^2^. In all groups, there were no major signs of chronic inflammation. However, lymphatic structures and leukocytes were noted mainly near scaffold strands. Additionally, non-specific, localized, low-grade granulomatose reactions were noted near localized scaffold strands. Overall, their minipig model was found to successfully regenerate large volumes of adipose tissue—suggesting that such a strategy could be applied for clinical applications. It might also serve as a way to differentiate such tissues in vitro as well for drug screening applications.

### 3.4. The Role of 3D-Printing for Nipple Reconstruction

**Figure 6:**
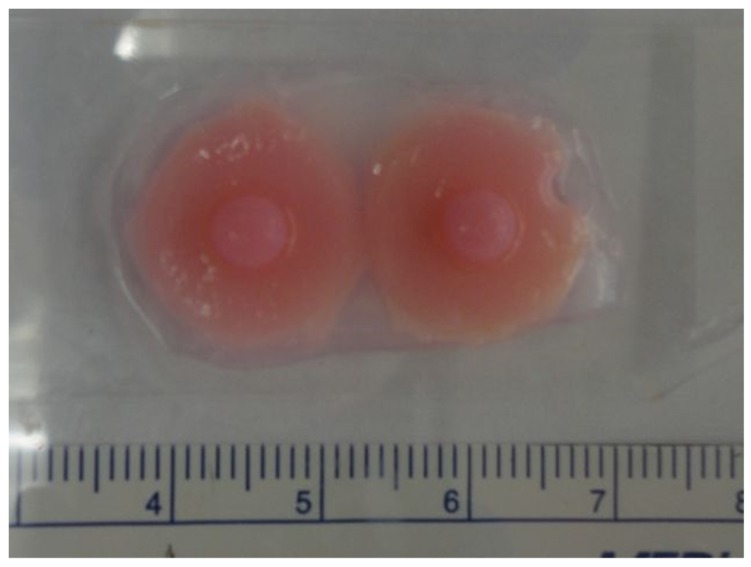
3D-bioprinted nipple prototype produced by TeVido Biosciences. Source: TeVido BioDevices.

Recreating the nipple-areola complex during breast reconstruction has been challenging. Studies show that nipple recreation is highly correlated with patient satisfaction and body image after breast reconstruction [53]. Current techniques, including local flaps, and pigmented skin grafts have unpredictable long-term outcomes in terms of neo-nipple projection, color, size, shape and texture, and the advantages of a 3D-bioprinted nipple areola complex have been suggested [31,54,55]. We recommend the reader look to the 2019 review by Khoo et al. for an in-depth review of the current challenges faced in nipple reconstruction [55]. While no 3D-bioprinted nipple has been reported to date, TeVido BioDevices, a Texas based company, is developing a 3D-bioprinted nipple areola complex graft. Their design aims to use a patient’s own fat tissue and cells to regenerate a projection build of pre-vascularized adipose grafts, with a custom-colored pigmentation to match the existing nipple, all in a single product for a plastic surgeon to apply in one sitting (Figure 6). 

NovoThelium is another pre-clinical startup, founded in San Antonio in 2017. They are developing a decellularized nipple reconstruction model. This method uses a patent-pending decellularization process to remove cells and DNA from nipple tissue provided by cadavers, leaving the intact 3D extracellular matrix to be grafted onto patients and repopulated by the patient’s own cells. They are also seeking to regenerate pigmentation using cell signaling cues specific to the nipple to promote melanocyte survival and restore sensation using guided nerve growth technology. Both of these start-ups are taking a novel approach to generate replacement nipple tissues, though much work remains to be done in this area. 

## 4. Discussion

Overall, the studies reviewed here show the promise of 3D printing for generating replacement breast tissue, but also that much work is still required to validate these tissues for clinical applications, as well as for drug screening applications. Melchels et al. showed that 3D-printed designs produce better outcomes in shape and symmetry in their three-patient study [49]. This argument should similarly apply to 3D-bioprinted nipple areola complexes in the future, and it will enable personalization of bioprinted tissues. Meanwhile, Pati et al. have shown that adipose derived ECM is sufficient to induce adipocyte differentiation from ASCs, and to promote blood vessel ingress from host tissues in a mouse model [51]. They also spearheaded the use of a dual bioink, consisting of softer dECM to promote regeneration, surrounded by a stronger synthetic PCL framework to provide support. While promising, their study was limited by size, generating a 5 cm^3^ domed construct, while the mean implant size for an A-cup mastectomy patient is 295.3 ± 66.6 mL [56], suggesting that these tissues might be more suitable for drug screening applications until appropriate scaling up can be performed. Chhaya et al. generated more clinically relevant volumes of tissue (30 mL) by using a synthetic PCL framework to first induce host blood vessel infiltration, followed by lipoaspirate injections containing a mixture of ECM components, adipocytes and ASCs to enhance adipogenesis [52]. This method of vascularizing the construct before introducing the cellular component resulted in the largest volume of tissue regeneration by 3D-bioprinting seen to date. However, it is of note that the use of animal models in these studies may have poor predictive value of the adipogenic capabilities found in humans, as illustrated by the studies by Findlay et al., wherein an impressive 80 mL of regenerated adipose tissue was seen in their large animal study, whereas their human trial produced mainly fibrotic tissue [27]. This may be due to the failure of animal models to recapitulate the complexity of human adipogenesis and inflammation [27,57,58]. In the meantime, such engineered tissues could be used to screen potential drug targets for toxicity to breast tissue. 

**Figure 7:**
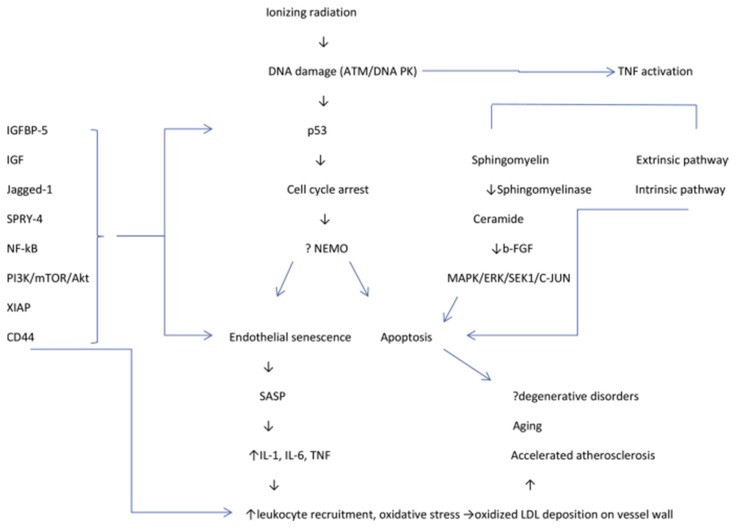
Proposed interplay between apoptosis and endothelial senescence, and its implications for pathogenesis, taken from [62]. ATM = ataxia telangiectasia mutated; b-FGF = basic fibroblast growth factor; ERK = extracellular signal-regulated kinase; IGF = insulin-like growth factor; LDL = low-density lipoprotein; MAPK = mitogen-activated protein kinase; SASP = senescence-associated secretory phenotype; SEK1 = stress-activated protein kinase 1; TNF = tumor necrosis factor. This figure is being reprinted under a Creative Commons BY 4.0 license.

Secondly, moving towards a more representative pre-clinical model that is capable of capturing the post-treatment tissue environment would be advantageous in predicting the regenerative capabilities of breast cancer survivors. Individuals who have had breast cancer treatment will have been irradiated at the site of tissue loss, leading to an altered tissue pathology in the remaining non-neoplastic breast tissue, wherein vascularization and regeneration are compromised [59,60,61,62,63]. Indeed, a host of cellular changes resulting in fibrosis, vascular thickening, chronic inflammation, fat necrosis, and abnormal angiogenic responses have been attributed to radiation treatment. Of these changes, the most often observed is that of atypical endothelial cells within the terminal duct lobular units, accompanied by vessel stiffening and degeneration [59,60,61]. A second characteristic event is the thickening of arteries and arterioles, sometimes manifesting in vessel occlusion. The third event, less often seen (24–36% incidence), is fat necrosis, likely due to the damaged blood vessels resulting in ischemia and inflammation [61,63,64]. The existence of these pathologies argues that the post-treatment breast tissue environment is severely altered in its ability to support or regenerate fat tissue or blood vessels. The reason for this altered pathology seems to lie in the actions of endothelial cells. When subjected to irradiation, endothelial progenitor cells undergo cell cycle arrest and apoptosis, while well-differentiated endothelial cells undergo senescence (Figure 7). This senescence, termed a “senescence-associated secretory phenotype” is accompanied by inflammatory cytokines, chemokines and extracellular proteases. Furthermore, irradiation triggers the generation of reactive oxygen species (ROS) in these cells, which in turn disrupt regular mitochondrial processes, further contributing to their degeneration and premature aging [62]. Together, these actions provide an environment that is hostile towards regeneration, and indeed, in a study of microvascular endothelial cells, irradiation was seen to promote a profibrogenic phenotype expressing excessive connective tissue growth factor (CCN2), collagen type III (COL3A1), plasminogen activator inhibitor I (PAI-1), and ɑ-smooth muscle actin (ɑ-SMA) [62].

Given the disadvantages outlined in the Table 1 for the current 3D printing techniques, extrusion-based 3D-printing remains the most feasible technique for bioprinting at this time. Specifically, UV toxicity, limited scalability, and being limited to non-complex architectures are all factors that ultimately preclude the advancement and use of bioprinting in the majority of human organs and structures. Extrusion-based technique needs to be further improved in resolution to reach micro-vessel size, ideally reaching at least 5 µm resolution. Another of the pitfalls of extrusion-based technique is its slow speed, which ultimately limits scalability and its ability to meet with current demands for tissue and organ replacement. Given that extrusion-based techniques is the most ideal option for the majority of target tissues or organs at this time, further advances are required to improve speed and resolution to become more effective. Laser-assisted techniques offer nearly all the same advantages as extrusion-based, but with higher resolution—reaching that of micro-vessels—and medium speed. Unfortunately, laser-assisted techniques also have limited scalability, and at a much greater expense than extrusion-based techniques. Furthermore, the laser-assisted method does not have the ability to print multiple bioinks within a structure during the fabrication process in contrast to the extrusion-based method, and so if the costs of laser-assisted bioprinting can be reduced and advances are made towards increasing scalability, this technique may be ideal for simple tissues and organs.

The safety and biocompatibility of the breast tissue models discussed in the results section are promising. PCL and poly(ᴅ,ʟ)-lactide polymer are FDA approved, biodegradable thermoplastic polymers, and the dECM used by the Pati group can be derived autologously. The polymers mentioned are well established in the biomedical field for applications such as wound repair and regeneration [65]. Although PCL is known to be safe for implantation, it offers poor cell adhesion properties, making it difficult to achieve long-term high cell viability and tissue formation within a printed structure [65]. Autologous dECM theoretically offers the benefit of completely evading a foreign body response, while taking advantage of naturally existing biophysical cues that promote tissue regeneration [52]. Additionally, dECM may offer patients the comfort of knowing they are receiving components of their own natural tissue to regenerate what was lost. 

The development of a human in vitro model for testing these post-breast cancer environmental conditions would benefit the discovery and validation of novel breast regeneration strategies. Such strategies could take advantages of new bioinks and 3D printing methods, along with new stem cell technologies like induced pluripotent stem cells. Overall, this review demonstrates the promise of 3D printing breast tissues and its potential for further applications. 

## Figures and Tables

**Table 1:** The advantages and disadvantages of the four main 3D-printing techniques.

Technique	Advantages	Disadvantages
Extrusion-based	Utilize high cell densities, high viscosity bioinks, print multiple bioinks simultaneously	Moderate resolution (~100 µm), slow speed, reduced cell viability secondary to shear stress, moderate cost
Advances: Melt writing Core-shell Microfluidic	Precise High cell viability High cell viability and resolution	Requires cell seeding post-print, moderate resolution (~100 µm) -- --
Inkjet	High resolution (50 µm), fast speed, low cost, widely available	Not suited for viscous bioink, limited to non-complex architecture, reduced cell viability secondary to heat or shear stress, low cell density
Laser-assisted	No shear stress, viscous or solid bioink compatibility, high resolution (1–50 µm)	Reduced cell viability secondary to heat, medium speed, limited scalability, high cost
Stereolithography	No shear stress, high resolution (3–300 µm), high cell viability, fast speed, low cost	Require photo-curable bioink, UV toxicity to cells, poor hollow-structure capabilities
